# Inactivation of *Clostridium perfringens* C1 Spores by the Combination of Mild Heat and Lactic Acid

**DOI:** 10.3390/foods11233771

**Published:** 2022-11-23

**Authors:** Tingting Lin, Huan Bian, Zhilan Sun, Xinxia Wang, Fang Liu, Daoying Wang

**Affiliations:** 1 Jiangsu Key Laboratory for Food Quality and Safety-State Key Laboratory Cultivation Base, Ministry of Science and Technology, Nanjing 210014, China; 2 Institute of Agricultural Products Processing, Jiangsu Academy of Agricultural Sciences, Nanjing 210014, China; 3 Key Laboratory of Cold Chain Logistics Technology for Agro-Product, Ministry of Agriculture and Rural Affairs, Nanjing 210014, China

**Keywords:** *C. perfringens*, spore, lactic acid, mild heat, DPA

## Abstract

*Clostridium perfringens* is a major pathogen causing foodborne illnesses. In this experiment, the inactivation effects of heat and lactic acid (LA) treatments on *C. perfringens* spores was investigated. Heat treatment (80 °C, 90 °C and 100 °C), LA (0.5% and 1%), and combined LA and heat treatments for 30 and 60 min were performed. Residual spore counts showed that the count of *C. perfringens* spores was below the detection limit within 30 min of treatment with 1% LA and heat treatment at 90 °C. Scanning electron microscopy and confocal scanning laser microscopy results showed that the surface morphology of the spores was severely disrupted by the co-treatment. The particle size of the spores was reduced to 202 nm and the zeta potential to −3.66 mv. The inner core of the spores was disrupted and the co-treatment resulted in the release of 77% of the nuclear contents 2,6-pyridinedicarboxylic acid. In addition, the hydrophobicity of spores was as low as 11% after co-treatment with LA relative to the control, indicating that the outer layer of spores was severely disrupted. Thus, synergistic heating and LA treatment were effective in inactivating *C. perfringens* spores.

## 1. Introduction

*Clostridium perfringens* is a Gram-positive anaerobic bacterium widely present in the environment and can be found in the air, soil, water, and human and animal intestines [1,2]. *C. perfringens* can produce metabolically dormant spores that are more resistant to various lethal factors, such as heat, chemicals, radiation, osmotic pressure, sodium nitrite, and pH than vegetative cells [3]. The spores have complex structures that make them resistant to high temperatures, high pressure, desiccation, and irradiation [4]. Therefore, killing them through conventional sterilization techniques is difficult. Once conditions are suitable, the spores can multiply rapidly and produce toxins that cause food spoilage and deterioration, leading to foodborne diseases [5]. *C. perfringens* isolates can be classified into one of 5 types (A to E) according to their ability to produce toxins into α-, β-, ε- and iota-toxins. *C. perfringens* isolates produce *C. perfringens* enterotoxin (CPE), an important human gastrointestinal pathogen that causes *C. perfringens* type A food poisoning and non-food sources food poisoning gastrointestinal disease [1]. CPE is only produced during enterosporation [3]. Vacuum-packed food in markets remains susceptible to these problems after a certain period of storage mainly because conventional sterilization technologies cannot inactivate residual spores or spores in contaminated food; these spores germinate and grow under suitable conditions, becoming the main food spoilage bacteria [6].

Despite the efforts to find new sterilization technologies that have less impact on food properties, heating is still considered the most effective technique because of the reduced lethality of non-thermal techniques in terms of bacterial spore inactivation [7]. However, the heating sterilization at high temperature (121–135 °C) and high pressure can negatively affect flavor, color, and quality due to protein deformation and nutrient breakdown of food [8]. Lactic acid (LA) is a natural organic acid that has long been designated by the U.S. Food and Drug Administration as a safe product “Generally Recognized As Safe” (GRAS) for meat products, and it has no limit in the acceptable daily intake for humans [9]. The scientists studied the effect of heat treatment intensity on the ability of citric acid or LA to prevent the viability of *Bacillus coagulans* spores after storage, and they found that organic acid could enhance the ability to inactivate *B. coagulans* spores by heat treatment [10]. Sun et al. also reported that LA and acetic acid have a synergistic effect on the inhibition of acid-tolerant spore-forming spoilage bacteria [11]. *C. perfringens* is one of the most common foodborne pathogens, so how to establish an effective method to inactivate this spore in food is still a problem to be solved. So far, Velugoti et al. reported the chemicals of buffered calcium, potassium, and sodium citrate can inhibit the germination and growth of *C. perfringens* spores [12]. Akhtar et al. formulated an inactivation strategy of *C. perfringens* spores using the combination of low pressure (100–200 MPa) and high temperature [13]. The combination of LA and heat treatment to inactivate *C. perfringens* spores was rarely studied, so the effect of LA and mild heat treatment was evaluated in this study.

In this experiment, the effect of LA (0.5%, 1%), mild heat treatment and their combination to inactivate *C. perfringens* spores was studied. The possible mechanism of inactivation of *C. perfringens* spores by the combination treatment of LA and mild heat was further investigated by measuring the DPA content of treated spores, the hydrophobicity of the outer structural protein and the changes in the zeta potential of the amino acid charge of the outer protein.

## 2. Materials and Methods

### 2.1. Bacterial Strains and Spore Preparation

*C. perfringens* strain C1 was isolated from chilled chicken meat and identified through 16S rRNA sequencing. *C. perfringens* was cultured in tryptone glucose yeast medium (Qingdao Gaoke Park Haibo Biotechnology Co., Ltd., Qingdao, China) in an anaerobic culture tank (Mitsubishi Gas Anaerobic Culture Tank, Chiyoda District, Tokyo, Japan) for 24 h. An anaerobic gas production kit (Haibo Biotechnology Co., Ltd., Qingdao, China) was used in this study.

*C. perfringens* spores were prepared used the methods described by Juneja et al. [14] with minor modifications. The cultured *C. perfringens* (0.1 mL) was added to 10 mL of fluid thioglycollate medium (FTG, Haibo Biotechnology Co., Ltd., Qingdao, China) and activated by heating in a water bath at 75 °C for 20 min. The *C. perfringens* was inserted into the FTG to make the *C. perfringens* proliferate and cultured anaerobically for 24 h. In the medium, tryptone and yeast extract provided carbon and nitrogen sources, vitamins, and growth factors. After activation, the cultured bacteria were rapidly cooled and incubated for 18 h anaerobically. Then, 1 mL of this culture was further aspirated into 10 mL of FTG broth and incubated at 37 °C for 4 h. The cultures were further incubated in modified Duncan-Strong broth for 3 days anaerobically. The spores were collected through centrifugation at 16,000× *g* and 4 °C for 20 min and re-suspended in 0.1% sterile peptone water. The concentration of the prepared spores (~4.2 log CFU/mL) was determined after incubation on tryptone sulfite-cycloserine agar (TSC, Qingdao Haibo Biotechnology Co., Ltd., Qingdao, China) anaerobically for 24 h. The spores were stored at 4 °C until use.

### 2.2. LA and Heat Treatment

The *C. perfringens* spores were subjected to heat treatment (80 °C, 90 °C, and 100 °C), LA treatment (LA, 0.5%, and 1% (*V*/*V*)), and combined LA and heat treatments for 30 and 60 min. The spores were heat-activated at 75 °C for 20 min and then rapidly cooled to room temperature [15]. Then, 0.5% and 1% LA solutions were prepared by diluting 85% LA (Tianjin Comio Chemical Reagent Development Center) in sterile deionized water, respectively. Heat treatment was carried out in a constant temperature water bath. For combined LA and heat treatments, 0.5 mL of *C. perfringens* spores at a concentration of 4.2 log CFU/mL were added to 4.5 mL of different concentrations of LA solution for 30 min. The solutions were heating at different temperatures for 30 and 60 min. Finally, 0.5 mL of 4.2 log CFU/mL of *C. perfringens* spores were added to 4.5 mL of 0.85% NaCl and left at room temperature as a negative control.

### 2.3. Scanning Electron Microscopy

Scanning *electron* microscopy (SEM) was used in visually observing the degree of cell damage in *C. perfringens* spores after different treatments [16]. The spores were treated with 1% LA, heated at 90 °C, and subjected to treatment with 1% LA and heat at 90 °C for 30 min according to the method listed in 2.2. The samples after different treatment were centrifuged at 5000× *g* for 10 min to obtain the precipitate, respectively. The samples used for SEM analysis were prepared according to previous paper [17]. The obtained precipitates were suspended in 2.5% (*v*/*v*) glutaraldehyde, fixed at 4 °C for at least 12 h, dried, and placed in 1% (*v*/*v*) osmolytic acid for 90 min [18,19]. After gradient dehydration, the samples were sprayed with gold and observed through SEM (EVO-LS10, Zeiss, Oberkohen, Germany) at 5000× magnification.

### 2.4. Confocal Laser Scanning Microscopy (CLSM)

The permeability of cell membranes of *C. perfringens* spores subjected to LA and heat treatment was analyzed by CLSM according to previous papers [18,19]. The spores were treated with 1% LA, heated at 90 °C, and subjected to treatment with 1% LA and heat at 90 °C for 30 min according to the method listed in 2.2. The samples after different treatment were centrifuged at 5000× *g* for 10 min at 4 °C to obtain the precipitate. Each precipitate was added in 1 mL of 0.1 mol PBS, and was stained with 3 µL staining solution for 30 min protected from light in LIVE/DEAD BacLight viability Kit (Molecular Probes; Life Technologies, Eugene, OR, USA). CLSM images were observed with Leica Ultra View VOX CLSM (Leica Microsystems, Ltd., Wetzlar, Germany).

### 2.5. Determination of the Release Rate of DPA

The release of DPA in treated *C. perfringens* spores was determined through fluorescence spectrophotometry with slight modifications according to the previous method [20]. After the *C. perfringens* spores were treated according to the method in Section 2.2, they were centrifuged at 5000× *g* and 4 °C for 5 min. The supernatant was collected and mixed with TbCl_3_ solution (50 µM, pH 5.6). This lipid of 200 µL was dropped into a 96-well enzyme label plate for the assay. Fluorescence intensity was measured with a fluorescence spectrophotometer, and the excitation and emission light wavelengths were 270 and 545 nm, respectively. Untreated spores were included in the control group, and where the corresponding amount of spores was boiled for 1 h as the total DPA amount in all spores. The percentage of DPA release (DPA%) was calculated using the following equation:

(1) $$\mathrm{DPA}\%=\frac{\mathrm{Fd}}{\mathrm{Fi}}\times 100\%$$

where Fd is the amount of DPA released by the *C. perfringens* spore treatment group and Fi is the amount of initial total DPA.

### 2.6. Measurement of Particle Size and Zeta Potential

The particle size and zeta potential of *C. perfringens* spores after different treatments were determined with reference to the previously published methods [21,22]. *C. perfringens* spores need to be activated at 75 °C for 20 min and treated according the above. The cuvette was firstly rinsed, and then the samples were poured into the test cuvette to a height of about 2/3 of the cuvette, respectively. The frosted side of the cuvette faced itself and was placed in the clip slot to avoid contact with the non-frosted side. The sample was added to the potentiometric cell avoiding bubbles and do not touch the copper sheets on both sides with your hands, and the Nano-ZS particle size analyzer (Nano ZS 90, Malvern, UK) was operated to measure particle size and zeta potential.

### 2.7. Rate of Hydrophobicity Measurements

The hydrophobicity of the spore surface was determined through the phase separation of hexadecane and water according to the previous method [21,22] with slight modifications. The concentration of spores was kept consistent at 4.2 log CFU/mL for each experiment. The initial OD600 value of untreated or treated spore suspension represented by A0. Approximately 3 mL of spore suspension was vortex-mixed with 0.5 mL of hexadecane for 30 s and left to stand at room temperature for 15 min. The supernatant was aspirated, and the OD600 value (Af) was measured. The surface hydrophobicity of the spore suspension was calculated using the following equation:

(2) $$\mathrm{RSH}=\frac{A0-\mathrm{Af}}{A0}\times 100\%$$

### 2.8. Statistical Analysis

All the samples of this experiment had three replicates, and data were finally calculated as mean and standard deviation. The data were statistically analyzed using SPSS software. One-way analysis of variance and LSD tests were used to indicate significant differences (*p* < 0.05).

## 3. Results and Discussion

### 3.1. Inactivation of C. perfringens Spores

**Figure 1:**
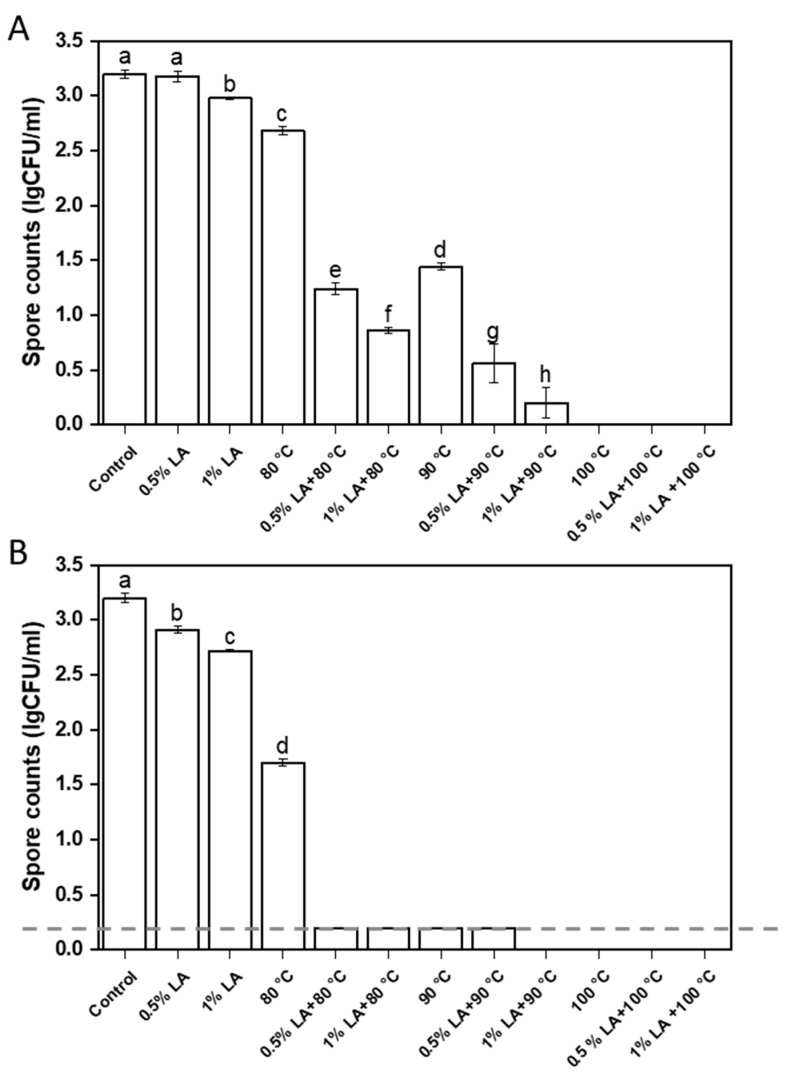
Counts of *C. perfringens* spores after different treatments for 30 min (**A**) and 60 min (**B**). Different lowercase letters indicated significant differences between treatments (*p* < 0.05); <0.2 log CFU/mL indicated absence of bacteria on the TSC plate but indicated micro-turbidity in the access FTG.

The effectiveness of different treatments in inactivating *C. perfringens* spores is illustrated in Figure 1. The antibacterial agents of 0.5% LA and 1% LA can only inactivate 0.02 and 0.2 log CFU/mL of *C. perfringens* spores within 30 min (Figure 1A). When the treatment time was extended to 60 min, they inactivated about 0.29 and 0.48 log CFU/mL of *C. perfringens* spores (Figure 1B). Heat treatments at 80 °C and 90 °C for 30 min inactivated *C. perfringens* spores at about 0.52 and 1.8 log CFU/mL, respectively. After pretreatment with 0.5% LA for 30 min, heat treatments at 80 °C and 90 °C inactivated *C. perfringens* spores of 1.93 and 2.64 log CFU/mL, respectively. After pretreatment with 1% LA for 30 min, heat treatments with 80 °C and 90 °C inactivated *C. perfringens* spores at 2.3 and 3 log CFU/mL, respectively. At the same temperature, the number of inactivated spores inactivated by combined LA and heat treatments was much higher than the totaled amounts in LA and heat treatments (*p* < 0.05).

As shown in Figure 1B, the number of *C. perfringens* spores decreased to less than 0.2 log CFU/mL after 60 min of heat treatment at 90 °C, and they were completely inactivated to zero after 30 and 60 min of heat treatment at 100 °C (Figure 1A). Pretreatment with 1% LA for 30 min followed by heat treatment at 90 °C for 60 min completely inactivated 3.2 log CFU/mL of *C. perfringens* spores. This result suggested that pretreatment with LA helped reduced the required temperature for heat treatment to completely inactivate *C. perfringens* spores.

*C. perfringens* spores were extremely resistant to heat, acid, and radiation [23,24] and can survive after the sterilization of meat products [25]. Wang et al. showed that more than 90% of *C. perfringens* spores are inactivated when incubated in water at 90 °C and 100 °C for 10 to 30 min [26]. Liang et al. found that combined treatment with 200 MPa and 1 mg/mL PGF at 80 °C for 20 min resulted in 8.6 log inactivation of *Bacillus subtilis* 168 and more than 5 log reduction of spores of *Clostridium perfringens* PA3679, respectively [27]. Evelyn et al. investigated the effect of simultaneous sonication and heating (TS, thermosonication) on spore inactivation in beef slurry. A 60 min TS procedure (24 kHz, 0.33 W/g) at 75 °C resulted in less than 1.5 log reduction of spores in *C. perfringens* NZRM 898 and NZRM 2621 [28]. Chen et al. [29] reported the inactivation of *E. coli* in organic broccoli subjected to combined LA and mild heat treatments, and in vitro survival kinetics showed a dramatic decrease in the number of cells. The decrease was undetectable after 120–135 s. Wang et al. showed that pathogens can be completely inactivated after exposure to LA [30]. In our experiments, LA almost broke the outer membrane and induced high protein leakage, and mild heating exacerbated the membrane damage caused by LA.

### 3.2. Scanning Electron Microscopy (SEM) Analysis

**Figure 2:**
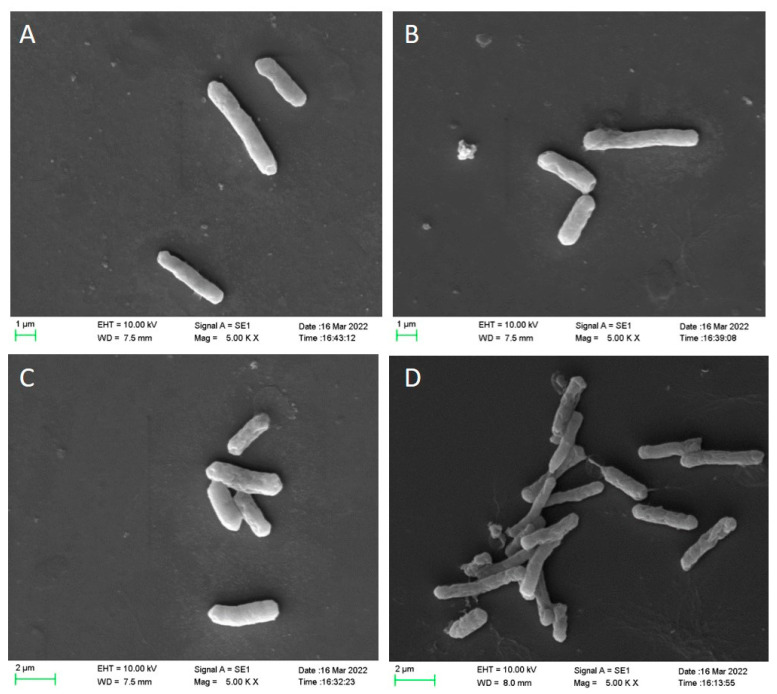
SEM images of C. perfringens spores after different treatments for 30 min. (**A**), control; (**B**), treatment of 90 °C; (**C**), treatment of 1% LA; (**D**), combined treatment (90 °C and 1% LA).

SEM was used in analyzing damage to the cell membranes of *C. perfringens* spores after different treatments. The result is shown in Figure 2. The surfaces of untreated spore-forming *C. perfringens* cells showed regular and intact rod-like morphology without obvious surface damage (Figure 2A). After treatment with 1% LA for 30 min, the surface of *C. perfringens* spores remained intact with slight wrinkles (Figure 2B). After heat treatment at 90 °C for 30 min, the surfaces of *C. perfringens* spores had slight fractures and folds (Figure 2C). A single round of 1% LA and heat treatment did not cause significant morphological changes in the cell membranes of *C. perfringens* spores. After treatment with 1% LA and heat at 90 °C for 30 min (Figure 2D), the *C. perfringens* spores were dried and nicked, indicating severe damaged. The adhesion between the spore coat and cortical layer was prevented by LA treatment alone, and combined treatments synergistically produced significant increase in mechanical damage, causing changes in spore morphology. This suggests that although organic acids have some effect on spores, and the overall effect is still inferior to that of the combined treatment. It has been documented that LA causes cell collapse or even fragmentation with distinct pits and gaps [30]. This is consistent with what we observed under scanning electron microscopy.

### 3.3. Confocal Laser Scanning Microscopy (CLSM) Analyses

**Figure 3:**
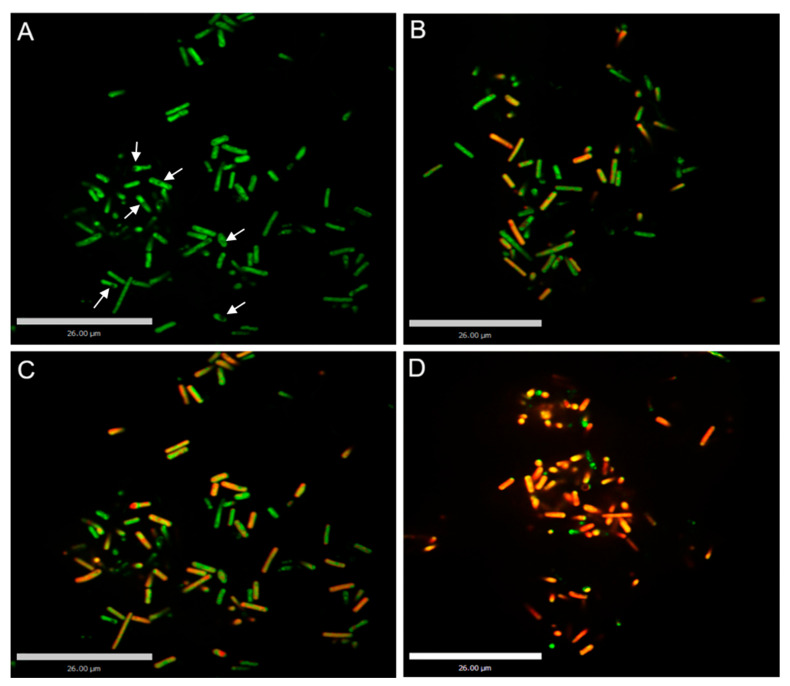
CLSM images of *C. perfringens* spores after different treatments for 30 min. (**A**), control; (**B**), treatment at 90 °C; (**C**), treatment with 1% LA; (**D**), combined treatment (90 °C and 1% LA). The white arrows referred to the spores.

Changes in the cell membrane permeability of *C. perfringens* spores after different treatments were analyzed through laser confocal microscopy. The results are shown in Figure 3. The untreated *C. perfringens* spores were almost bright green and the inner spores were black (Figure 3A). The green dye can inter into the cell membrane of intact *C. perfringens* spore cells but cannot inter into the thick spore membrane. The green color indicated that their cell membranes were intact. After treatment with 1% LA for 30 min, some *C. perfringens* spores turned yellow and red, indicating that some cell membranes were disrupted (Figure 3B). After treatment with heat at 90 °C for 30 min, a small proportion of *C. perfringens* spores turned yellow and red (Figure 3C). These images indicated that 1% LA or heat treatment alone did not increase the permeability of *C. perfringens* spores. After treatment with 1% LA at 90° C for 30 min, *C. perfringens* spores overwhelmingly turned yellow and red (Figure 3D), indicating that the cell membranes of the spores were severely disrupted. Ning et al. [31] found that the untreated bacterial cells with intact cell membranes emitted green fluorescence. After treatment with phenyl lactate, the cells fluoresced yellow or orange-red indicated that the cell membranes had different levels of disruption.

### 3.4. Determination of 2,6-Pyridinedicarboxylic Acid Release Rate (DPA%)

**Figure 4:**
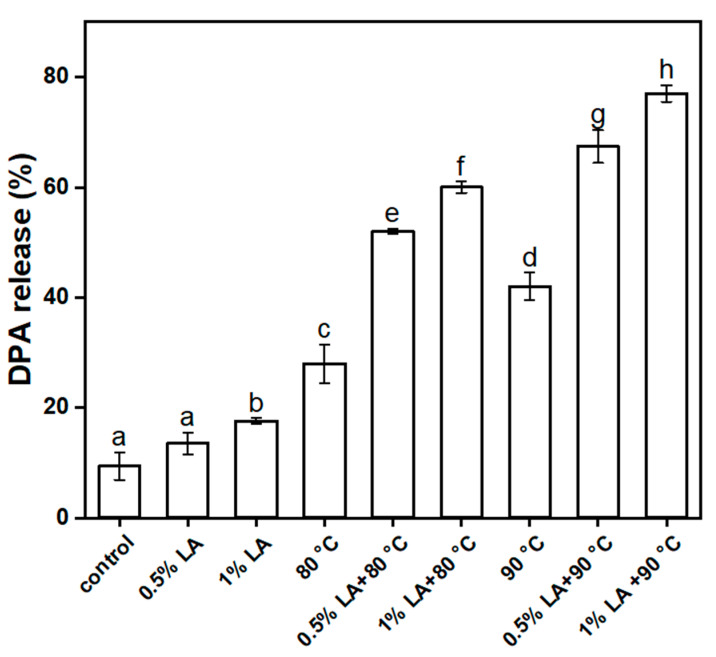
Plot of the changes in DPA release rate after different treatments for 30 min. Different lowercase letters indicate significant differences between treatments (*p* < 0.05).

The core layer of a spore contains a large amount of DPA, which is specific to the spore. After different treatments, DPA leaked out of the spores, indicating that the core layers of the spores were damaged. Changes in the DPA of the spores after different treatments are shown in Figure 4. Only 9.4% of DPA was released from the untreated spores. The DPA% values after treatment with 0.5% LA and 1% LA for 30 min were 13.5% and 17.6%, respectively. The DPA% values of the spores were 28% and 45% after being treated with heat at 80 °C and 90 °C for 30 min, respectively. This result showed that the effect of individual treatment DPA release was unsatisfactory, except for the group treated at 90 °C alone, in which it was slightly more pronounced. The DPA% values were 52% and 60% with 0.5% LA and 1% LA and heat treatment at 80 °C for 30 min. The concentrations of lactic acid treated separately at 0.5% and 1%, combined with heat treatment for 30 min at 80 °C, resulted in 52% and 60% release of DPA, respectively. After 0.5% LA and 1% LA and 90 °C heat treatment for 30 min, DPA% values were 67.4% and 77%, respectively. These results showed that the effect of the combined treatment was significantly stronger than that of a single treatment (*p* < 0.05). The combined treatment caused a gradual increase in DPA%, and the permeability of the inner membrane of the spore greatly increased or even collapsed due to the inactivation of structural proteins and subsequent release of DPA. Bevilacqua et al. [32] reported the inactivation of *Alicyclobacillus acidoterrestris* spores under different stress conditions (pH, temperature of 70 °C, hydrogen peroxide, p-coumaric acid, lysozyme) and the release of DPA, and the results confirmed that the DPA release from spores was partially related to their inactivation. Fan et al. investigated the synergistic inactivation and mechanism of *Bacillus subtilis* spores using combined heat and ultrasound treatment and found the release of DPA from spores after treatment and the change in equilibrium density between the untreated and treated groups. This study concluded that the release of DPA from *B. subtilis* spores occurred mainly after spore death [16].

### 3.5. Measurement of Particle Size and Zeta-Potential

**Figure 5:**
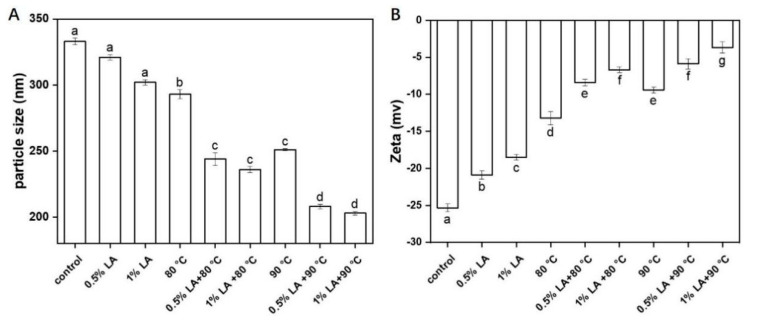
Changes in *C. perfringens* spore particle size (**A**) and zeta potential (**B**) after different treatments for 30 min. Different lowercase letters indicate significant differences among treatments (*p* < 0.05).

The particle sizes of the spores after different treatments are shown in Figure 5A. Whether the outer proteins of *C. perfringens* spores were damaged was determined by measuring the particle sizes of the spores. Untreated spores reached a particle size of 333 nm, and after treatment with 0.5% LA and 1% alone, particle size values of 323 and 301 nm were obtained. The particle sizes of *C. perfringens* spores were 293 and 255 nm after heat treatment at 80 °C and 90 °C for 30 min, respectively. The single treatments had little effect on particle size. After the treatment with 0.5% or 1% LA at 80 °C for 30 min, the spore particle sizes were 244 and 232 nm, respectively. After treatment with 0.5% LA or 1% LA at 90 °C for 30 min, the spore particle sizes were 208 or 203 nm, respectively. Compared with a single treatment (*p* < 0.05), the combined treatments resulted in a smaller spore particle size, indicating that the combined treatment disrupted the outer proteins of *C. perfringens* spores and was more effective than a single treatment. Fan et al. (2019) [16] reported the inactivation mechanism of ultrasonic synergistic thermal inactivation of *B. subtilis* spores, and spore particle size decreased after combined ultrasound and heat treatments at 80 °C, and their particle sizes decreased to a considerably larger extent than those of spores treated with combined ultrasound and heat treatment at 70 °C. LV et al. [21] showed the reason for the change in ultrasound on spore particle size is that the outer layer proteins of the spores were damaged during ultrasound treatment, resulting in a reduction in spore particle size. Pizarro-Guajardo et al. [33] found that the exospore of the spore is a balloon that occupies a large volume and the apparent reduction in size is caused by the detachment of the exospore.

Zeta potential depends on the charges of amino acids in the outer protein layers of spores. If the outer layer structure is disrupted, zeta potential decreases. As shown in Figure 5B, the absolute values of zeta potential for treatment with 0.5% LA, 1% LA, 80 °C, and 90 °C decreased to −20.1, −18.5, −13.2, and −8.5 mv, respectively, compared with those of the control (*p* < 0.05), whereas the absolute values of zeta potential for 0. 5% LA, 1% LA, and 30 min of heating at 80 °C were −8.4, −6.7, and −5.6, and −3.7 mv absolute values of zeta potential for 0.5% LA, 1% LA and 30 min of heating at 90 °C. Clearly, a combination of LA and heat treatment was more effective, especially treatment with 1% LA at 90 °C for 30 min. This result indicated that the external structural proteins of the *C. perfringens* spores were disrupted during treatment, leading to decreases in the surface areas of the spores and thus decreases in the absolute values of zeta potential. Fan et al. [16] proposed that the absolute value of the zeta potential of spores treated with combined ultrasound and heat treatments decreased and the outer structural proteins of the spores were damaged during the combined treatment. The strength of mutual repulsion between individual spores decreased with the absolute value of zeta potential [21]. Chen et al. [34] found that untreated spores had the highest absolute zeta potential, which decreased with increasing treatment time. In fact, solutions with high absolute zeta potential are considered to be more stable.

### 3.6. Hydrophobic Analysis

**Figure 6:**
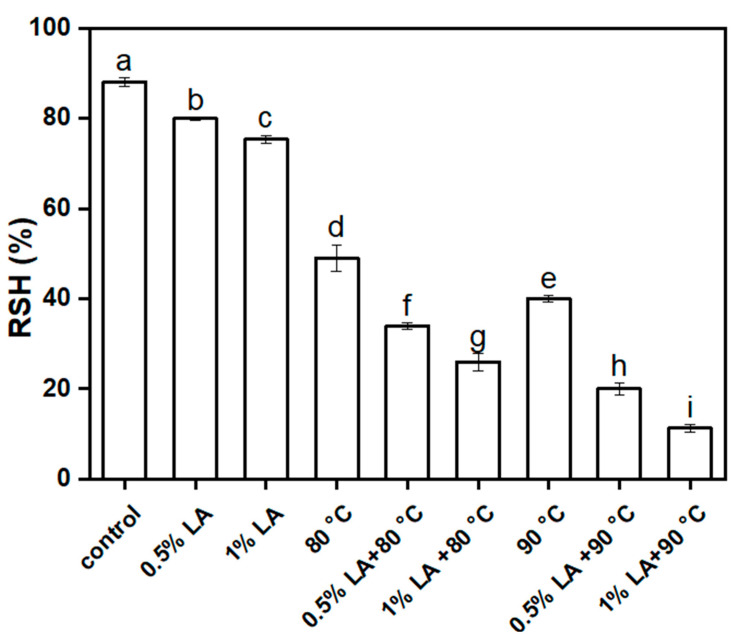
Changes of spore hydrophobicity after different treatments for 30 min. Different lowercase letters indicate significant differences between treatments (*p*< 0.05).

The outer structure of the spore had 60–70 different proteins that are importantly linked to spore structure assembly, spore resistance, and surface property exertion. Disruption or inactivation of the outer structure proteins of a spore changes surface properties, such as hydrophobicity and reduction in the absolute value of the zeta potential. The hydrophobicity of *C. perfringens* spores after different treatments is shown in Figure 6. Untreated spores had 88% hydrophobicity. After treatment with 0.5% LA and 1% LA for 30 min, the hydrophobicity of the spores decreased from 88% to 80% and 75%, respectively (*p* < 0.05). The hydrophobicity of the spores decreased from 88% to 49% and 40% after heat treatment at 80 °C and 90 °C for 30 min, respectively. These results indicated slight decrease in the hydrophobicity of the spores under a single treatment. The hydrophobicity of the spores decreased from 88% to 34% and 26.7% after treatment with 0.5% LA at 80 °C and 1% LA at 80 °C for 30 min, respectively. The hydrophobicity of the spores decreased from 88% to 20% and 11.3% after treatment with 0.5% LA at 90 °C and 1% LA at 90 °C for 30 min. This result indicated that the combined treatment with LA and heat treatment resulted in the low hydrophobicity of the spores. Adhesion and hydrophobicity of the spore surface are influenced by the protein composition of the spore coat [22,35]. Kutima and Foegeding noted that the hydrophobicity of waxy spores decreased when the spore coat was removed by chemical treatment [36]. The reason for this decrease in our hydrophobicity may be due to the fact that LA has previously disrupted the spore coat, resulting in a decrease in hydrophobicity of the spores.

## 4. Conclusions

In this study, the combination of LA and mild heat treatment achieved better effect to inactivate *C.perfringens* spores than single treatment. The effect of mild heat treatment is enhanced by the combined use of LA. The combined treatment caused a gradual decrease in the amino acid charge of the outer protein, and damaged the outer layer structure of the spore. The DPA, the core material inside the spore, was largely released after the combined treatment, indicating that the inner membrane of the spore was seriously damaged and its permeability was increased. The combination of LA and mild heat treatment can be a potential method to inactivate the spores in food products which cannot be sterilized at high temperature.

## Data Availability

The data presented in this study are available on request from the corresponding author.
